# Supplementary material for: Growth Performance and Adaptability of European Sea Bass (Dicentrarchus labrax) Gut Microbiota to Alternative Diets Free of Fish Products
Source: Microorganisms. 2020 Sep 3;8(9):1346. doi: 10.3390/microorganisms8091346 (PMC7565124; doi:10.3390/microorganisms8091346)
Supplement: Supplementary file 1 [file microorganisms-08-01346-s001.zip › microorganisms-908200-Sup-corrected.pdf]

SUPPLEMENTARY MATERIALS

Supplementary figures

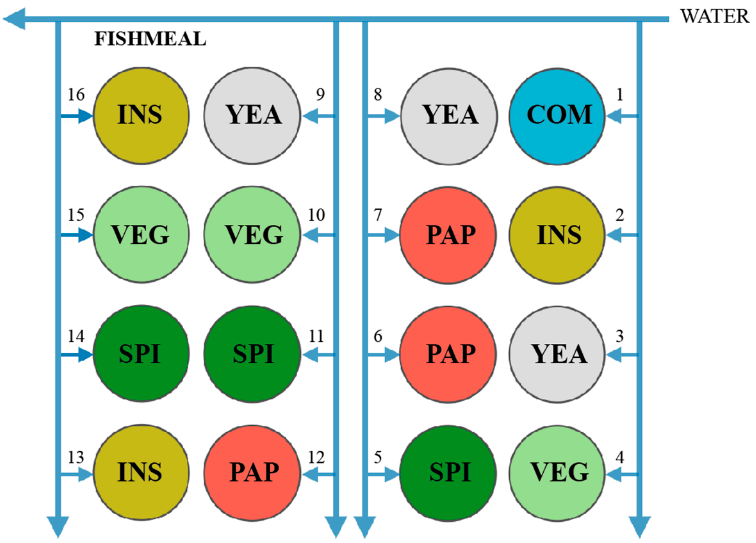

Figure S1. Experimental setup of the recirculating aquaculture system with random assignment of each diet to the tanks. There were 53 fish per tank and 3 tanks per fish feed treatment. See Figure 1 for corresponding diet descriptions.

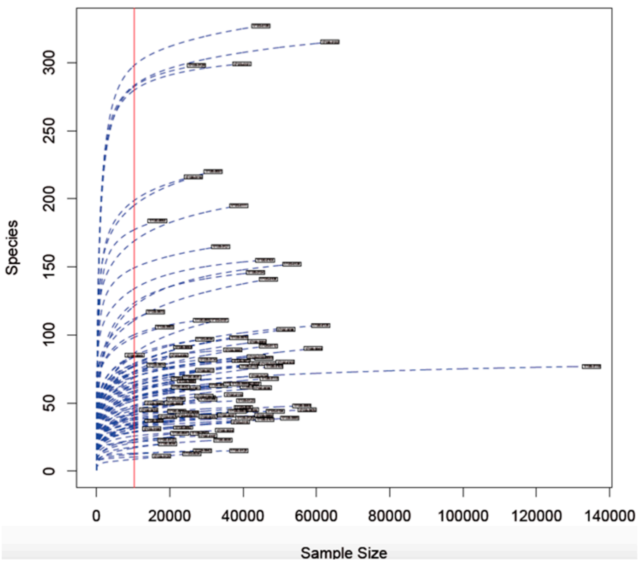

Figure S2. Rarefaction curves for each sea bass intestinal sample fed different diets. Rarefaction curves were assembled showing the number of operational taxonomic units defined at a 97% sequence similarity, relative to the number of total sequences. The vertical dotted-red line represents the plateau threshold for all curves (established to 10422 reads).

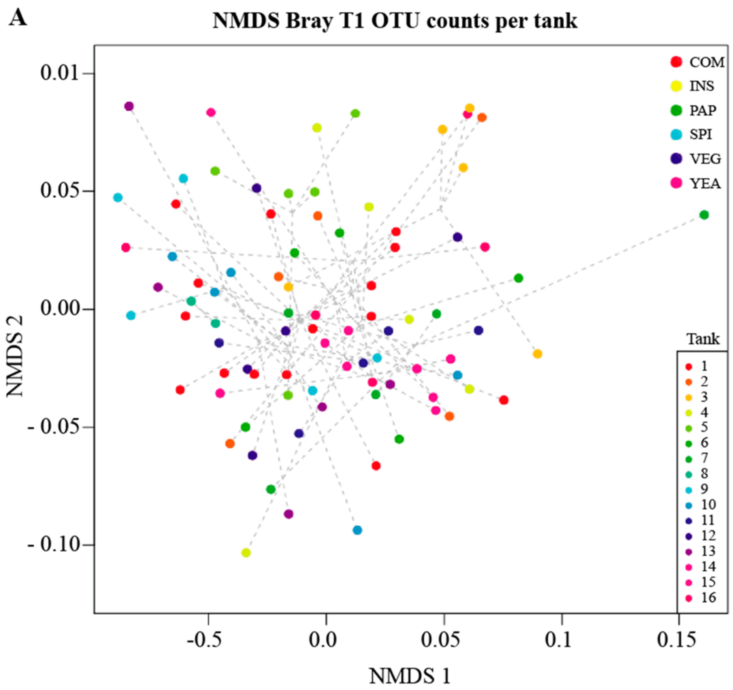

Figure S3. Non-metric multidimensional scaling plots based on Bray-Curtis dissimilarities showing the ordination between intestinal bacterial community of European sea bass from different aquaria at the end of the feeding trial (t = 93 days). Each dot represents an individual sample plot according to its microbial profile at OTU level (n = 12 to 16 fish/diet).

Supplementary tables

Table S1. Feed formulations for seabass (g/100 g feed) of commercial feed (COM), total vegetal feed (VEG) and experimental feeds (SPI, YEA, INS and PAP).

Ingredients	COM	VEG	SPI	YEA	INS	PAP
Fishmeal	25.3	0	0	0	0	0
Fish oil	9.3	0	0	0	0	0
Rich DHA algae meal		7.44	7.44	7.44	7.44	7.44
Insect meal					15	
Spirulina biomass			15			
Yeast protein fraction				15		
Processed animal proteins	22.2					15
Vegetable oils	6.2	14.6	14.3	13.85	11.6	13.75
Plant proteins	35.3	72.5	58.0	58.8	61.1	58.1
Rape seed lecithin		1.0	1.0	1.0	1.0	1.0
Monocalcium phosphate		1.80	1.54	1.89	1.50	2.19
Lysine 78%	0.43	1.20	1.20	0.58	0.92	0.95
DL-Methionine 98%	0.38	0.51	0.52	0.47	0.48	0.57
Threonine 98%	0.20	0.20	0.20	0.20	0.20	0.20
Vitamin premix	0.25	0.30	0.30	0.30	0.30	0.30
Vitamin C monophosphate 35	0.04	0.04	0.04	0.04	0.04	0.04
Mineral premix	0.25	0.30	0.30	0.30	0.30	0.30
Liquid choline	0.15	0.15	0.15	0.15	0.15	0.15

Table S2. Relative abundances (%) of the most prevalent phyla in the different dietary groups (A).

Phylum	VEG	INS	YEA	SPI	PAP	COM
Proteobacteria	49,76	56,20	56,57	53,00	49,31	59,33
Firmicutes	17,44	19,84	15,14	19,46	12,65	14,01
Actinobacteria	13,31	12,51	8,07	9,21	12,64	9,85
Bacteroidetes	2,85	6,28	6,31	6,11	6,64	6,01
Cyanobacteria	2,95	1,50	0,72	3,86	7,80	1,43
Verrucomicrobia	1,19	0,47	1,58	0,53	2,44	1,37
Fibrobacteres	0	0	0	0	0	1,32
Fusobacteria	0,34	0,71	0,10	1,81	0,22	1,16
TM7	0,57	0,00	0,26	0,28	0,07	1,10
OP11	1,70	0,36	5,13	2,45	3,85	1,09
GN02	3,82	0	0,84	0,09	0,23	0,49
TM6	2,15	0,36	2,15	0	0,89	0,07
Chlamydiae	1,58	1,28	0,78	0,55	0,50	0,41
Planctomycetes	1,15	0,11	1,43	1,95	1,09	0,77

Table S3. Relative abundances (%) of all identified genera in the different dietary groups. The relative abundance of identified genera for each sample as well as the corresponding average for each dietary group are presented in %.
